# Repositioning chair treatment procedure for cupulolithiasis: case report (with video)

**DOI:** 10.1007/s00405-024-08807-6

**Published:** 2024-07-13

**Authors:** Quentin Legois, Charles-Edouard Molinier, Pauline Nieto, Mathieu Marx

**Affiliations:** 1 Service d’ORL, Otoneurologie et ORL Pédiatrique CHU Toulouse Purpan, Toulouse, France; 2 Centre de Recherche Cerveau et Cognition, CNRS, 31300 Toulouse, France

**Keywords:** BPPV, Treatment, Cupulolithiasis, TRV repositioning chair, Case report

## Abstract

**Introduction:**

A cupulolithiasis of the lateral semicircular canal is an accumulation of otolithic debris at the level of the cupula of the same canal. Its pathophysiology generally generates a specific clinical presentation. This situation can be very disabling for the patient and tricky to treat for the clinician.

**Case report:**

The patient was a 70-year-old man with cupulolithiasis of the right lateral semicircular canal. We present here the conversion of cupulolithiasis to canalolithiasis using the Thomas Richard Vitton (TRV) repositioning chair, as well as the treatment of this canalolithiasis through a mechanical liberation maneuver.

**Conclusion:**

The results of manual therapeutic maneuvers for Benign Paroxysmal Positional Vertigo (BPPV) are generally good regardless of the type of BPPV. It can sometimes be more challenging to resolve an ageotropic-type BPPV of the lateral semicircular canal and mechanically-assisted maneuvers using a repositioning chair may be required. Faced with symptom resistance despite attempts at multiple liberatory maneuvers, clinicians must be able to reconsider their initial diagnosis and investigate other potentially more serious origins of these symptoms. The TRV chair can be a treatment option in the management of cupulolithiasis, especially in cases where classic reduction maneuvers do not always yield good results.

**Supplementary Information:**

The online version contains supplementary material available at 10.1007/s00405-024-08807-6.

## Introduction

The term “cupulolithiasis” was first used by Schuknecht in 1969 to describe positional paroxysmal vertigo originating from the deposit of minerals on the cupula of the posterior semicircular canal [1]. These mineral deposits can also occur in the cupulae of the lateral semicircular canals with a specific clinical presentation characterized by ageotropic nystagmus when the head is turned to the right or left in a bilateral supine position, and the presence of a null point when the patient turns the head very slightly toward the side of their cupulolithiasis [2]. The treatment of choice for cupulolithiasis of the lateral semicircular canal is the Gufoni maneuver [3]. Its efficacy at 24 h post-treatment was demonstrated in the geotropic and ageotropic forms of BPPV of the lateral semicircular canal (83.8% of success) compared to a sham maneuver (11.4% of success) in a double-blind randomized trial [4].

BPPV can, in certain specific indications, be treated with a repositioning chair such as the TRV chair, and the effectiveness of this treatment has been demonstrated [5, 6].

The management of this cupulolithiasis was recorded for educational purposes because cupulolithiasis-type BPPVs remain relatively uncommon, and the modalities of the therapeutic sequence (cupulolithiasis turns into canalolithiasis, then released) unrecognized. Moreover, the likelihood of obtaining a quickly satisfactory response to the Gufoni maneuver is also low [7].

We also wanted to demonstrate that there can be an alternative for cupulolithiasis cases resistant to table maneuvers.

## Case-report

The patient gave consent for the diffusion of their case report to the scientific community.

This 70-year-old patient was referred in our hospital department for positional rotatory vertigo. The patient had a history of BPPV, with the most recent episode occurring 7 months ago (BPPV of the right posterior semicircular canal).

In the days leading up to the visit, the patient had spent several hours working with his head in extension and hyperflexion while laying a floor.

Clinical examination revealed a positive bilateral Head Roll Test (HRT) with untiring ageotropic horizontal nystagmus, more symptomatic in left HRT. The null point (disappearance of nystagmus) was in a slight rightward rotation. Surprisingly, the Bow test and Lean test were negative.

A cupulolithiasis of the right lateral semi-circular canal was suspected. The classical diagnostic tests and therapeutic maneuvers were difficult to perform because patient’s reduced mobility due to intense nausea. It was then decided to reassess the patient and perform the maneuvers the next day using the TRV chair.

**Fig. 1:**
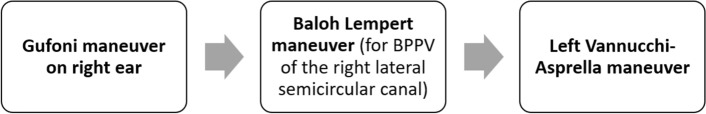
Diagram illustrating the therapeutic maneuvers used

On the following day, the clinical presentation remained similar. A Gufoni maneuver on the patient’s right ear were performed to convert the ageotropic horizontal positional nystagmus to a geotropic one (Fig. 1), wich would indicate the conversion from cupulolithiasis to canalolithiasis. Upon retesting, the nystagmus was indeed geotropic in bilateral HRT, and the patient was much more symptomatic in right HRT.

We proceeded with the mechanical Baloh Lempert maneuver adapted for BPPV of the right lateral semicircular canal (Fig. 1). Following this maneuver, the patient was significant improved, without any vertigo, and no nystagmus was observed in bilateral HRT.

We instructed the patient to perform a left Vannucchi-Asprella maneuver for three nights and reviewed him one week later (Fig. 1). One week after the treatment, the patient did not complain of any vertigo and was asymptomatic in bilateral HRT (without nystagmus).

## Discussion

The resistance of BPPV to liberatory maneuvers in the case of cupulolithiasis of the lateral semicircular canal is relatively common. One of the reasons of failure with table Gufoni maneuvers is that the magnitude and speed of execution of the maneuvers are sometimes insufficient [7]. The centrifugal force delivered by mechanical assistance would facilitate the easy detachment of adherent otolith [7].

Some clinicians combine Gufoni maneuvers with mastoid oscillations in the treatment of cupulolithiasis, and report more than 70% of success [8].

However, repeated failures of well-conducted maneuvers (whether manual or mechanically-assisted) should lead to reconsider the initial diagnosis. Indeed, other disorders/pathologies can present with similar clinical manifestations, like age-related degenerative disorders [9], or central positional nystagmus [10].

## Conclusion

This case report proposes an effective alternative to the Gufoni maneuver typically performed on a table, using a dedicated repositioning chair. Given the limited accessibility of this type of chair, it is recommended for use in specific cases such as failed table maneuvers, patients with limiting comorbidities (overweight, orthopedic disorders). However, caution is advised not to overlook ageotropic nystagmus of central origin, for which the use of the chair may have no apparent effect and could even be contraindicated in certain situations.

## Supplementary Information

Below is the link to the electronic supplementary material.

Supplementary file1 (MOV 61808 KB)

## Data Availability

Data transparent.
